# The distribution of burden of dental caries in schoolchildren: a critique of the high-risk caries prevention strategy for populations

**DOI:** 10.1186/1472-6831-6-3

**Published:** 2006-01-31

**Authors:** Paul A Batchelor, Aubrey Sheiham

**Affiliations:** 1 Dental Public Health, Department of Epidemiology and Public Health, University College London, 1-19 Torrington Place, London WC1E 8BT, UK

## Abstract

**Background:**

The 'high-risk approach' is a commonly adopted strategy recommended for the prevention of dental caries in populations. The scientific basis for the strategy has been questioned. The objective of this study is to assess the contribution that children identified at 'high-risk' made towards the total of new caries lesions over a 4-year period, by analysing the distribution of new lesions per 100 children.

**Methods:**

Data are from the National Preventive Dentistry Demonstration Programme (NPDDP) in the United States. The analyses identified the distribution of new carious lesions over a 4-year period in four groups of 7 year-old children who received differing preventive regimes.

**Results:**

The majority of new lesions occurred in those children classified at lowest caries risk at baseline. Irrespective of the preventive regime adopted and the initial caries levels, children classified as 'highest risk' contributed less than 6% of the total number of new lesions developing over 4 years.

**Conclusion:**

These findings challenge the basis for the adoption of a high-risk strategy.

## Background

A commonly adopted approach for the prevention of caries is the 'high-risk' strategy. For example, Messer [1] concluded that "... the need for, targeted prevention of dental caries for those at high risk has become apparent". The approach is based on three assumptions: first, those individuals with high future caries increments can be identified; second, measures taken to prevent the caries lesions are effective, and third, that those individuals belonging to sub-groups within a population who have previously experienced the highest levels of caries in the past will continue to do so in future. Indeed, any high-risk strategy aims to target those individuals at the greatest risk of future disease based on their current caries status or markers of disease.

From a public health perspective, what is important when deciding upon a preventive approach is what impact the measure adopted would have on the total dental health and disease burdens of the population as a whole. Even if an approach was highly accurate in predicting future caries development and the intervention was relatively successful in reducing caries in that group, the distributive features of new disease may make the proposed approach inappropriate. Even if a high-risk group had a far higher annual increment than the remaining sub-groups of the population, due to the underlying distributive properties of caries within a population, a far greater number of lesions are likely to occur in the low risk individuals because there are more of them. Batchelor and Sheiham [2] referred to this issue when examining caries distributions within a population. They outlined the limitations of adopting a 'high-risk' approach for the prevention of caries highlighting that any changes in the average caries experience within the population were not limited to specific sub-groups but occurred throughout the population. In addition, they found a mathematical relationship between the mean DMF score for a population and the prevalence of caries within that population. For a given mean DMF score the prevalence within the population could be defined and vice versa. The relationship was independent of both age and fluoride levels [2].

The problems of a high-risk strategy are increased by the low accuracy of methods used to identify the high-risk children. While the idea that an individual's future caries increment can be predicted from their past caries experience underpins the basis of caries risk assessment, the rigour of these measures to date is poor. Powell [3] and Zero et al. [4] reviewing the literature covering the use of indicators of risk found that the predictive validity of the models were heavily dependant upon the prevalence of caries and the characteristics of the population for which they were designed. Zero et al. [4] found that a single indicator gave as good results as more complex combinations of variables. This finding is in agreement with van Palenstein Helderman et al. [5] who, using longitudinal study data examining past caries variables, found that the gain in accuracy by including additional predictor variables was limited. Irrespective of their complexity, no predictive model is able to identify those individuals who will get the highest future caries increments. Hausen et al. [6] and Hausen [7] have highlighted the limitations of current methods used to identify high-risk individuals. Furthermore, even at a population level Poulsen and Scheutz [8] also recognised that a high-risk strategy could be challenged on the grounds of effectiveness. Examining the changes in dental caries experience in Danish children and adolescents over a ten-year period, they concluded that, if adopted, a high-risk strategy that was 40% effective would reduce the mean DMFS for the whole population by a mean of only one surface.

A major shortcoming of the high-risk approach is the failure to examine its impact on the overall number of new lesions within a population. Does a strategy targeting the high-risk group prevent more lesions for a population than a whole population strategy? Do 'low risk' sub-groups develop less new lesions than those with high caries levels? To answer the question, an analysis is required of the distribution of new caries lesions within a population as the baseline levels of caries increase.

Our critique of the high-risk strategy which we shall demonstrate in this paper is based on the concept that the largest "... burden of ill health comes more from the many who are exposed to low inconspicuous risk than from the few who face an obvious problem" [9]. The aim of this study is to assess whether 'high risk groups' of children accounted for a high percentage of new caries lesions in children. The objective was to analyse the distribution of new lesions per 100 7-year-old children in four populations with differing initial caries levels over a 4-year period.

## Methods

The data used were derived from the National Preventive Dentistry Demonstration Programme (NPDDP). The programme was an extensive project aimed at determining the costs and benefits of various types and combinations of school-based preventive dental care procedures. The project ran from 1976 to 1983, with the data used here being collected over the four-year period 1977–81. The project was conducted in 10 communities in the United States, 5 non-fluoridated and 5 fluoridated. The preventive regimes included fissure sealants, topical fluoride rinses, fluoride tablets, and school based oral health education programmes. The subjects in the trial were children in grades 1, 2 and 5 in the participating schools. From those selected as eligible to participate in the study, 82% responded positively. The data used in this study cover only those children. No attempt was made to assess the impact of non-respondents as the aim of the present study is fundamentally different from that of the original project. The background, organisation and results of the NPDDP programme have been reported elsewhere [10-13].

The children were categorised according to age, geographical location and preventive programme. The minimum number in each group exceeded 500 children. The mean 4-year caries increment was recorded for each of the sub-groups created according to the child's water fluoridation and sealant status. Group 1, formed by children who did not receive fluoridated water but who received sealants had an initial DMF-S score of 0.61; Group 2, whose water supply was not fluoridated and did not receive sealants, a DMF-S of 0.85; Group 3, who lived in fluoridated water supply areas but did not receive sealants, a DMF-S = 0.86 and Group 4, fluoridated and sealants, a DMF-S = 0.95. For the purposes of this study the four groups were followed over a 4-year period to assess the distribution patterns of new caries lesions. The data were analysed using SPSS Version 11 for Mac OS X.

## Results

**Figure 1:**
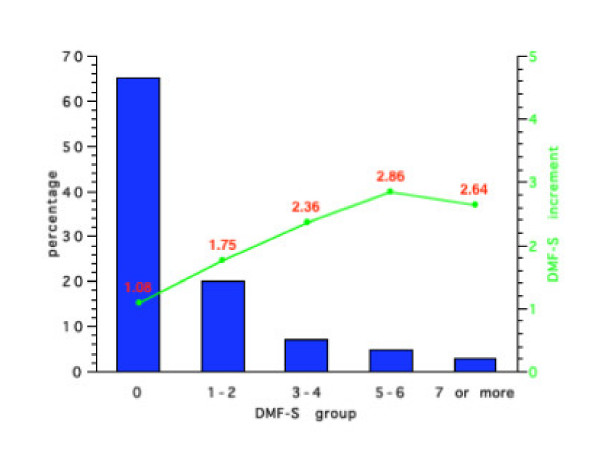
Initial percentage distribution and subsequent mean 4-year DMF-S increments in Group 1 (Initial DMF-S = 0.61).

**Figure 2:**
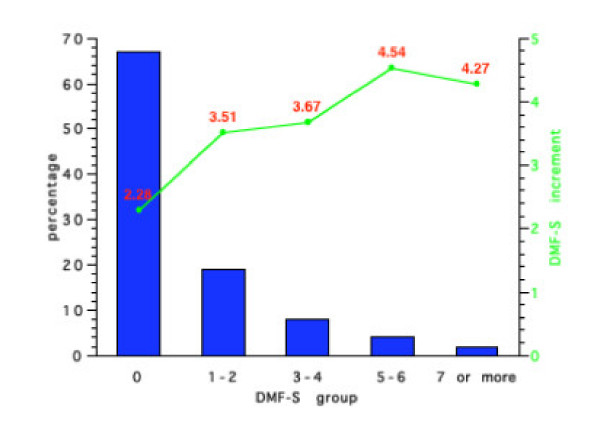
Initial percentage distribution and subsequent mean 4-year DMF-S increments in Group 2 (Initial DMF-S = 0.85).

**Figure 3:**
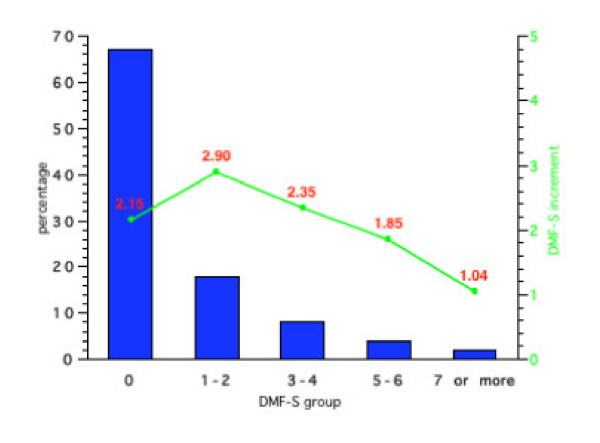
Initial percentage distribution and subsequent mean 4-year DMF-S increments in Group 3 (Initial DMF-S = 0.86).

**Figure 4:**
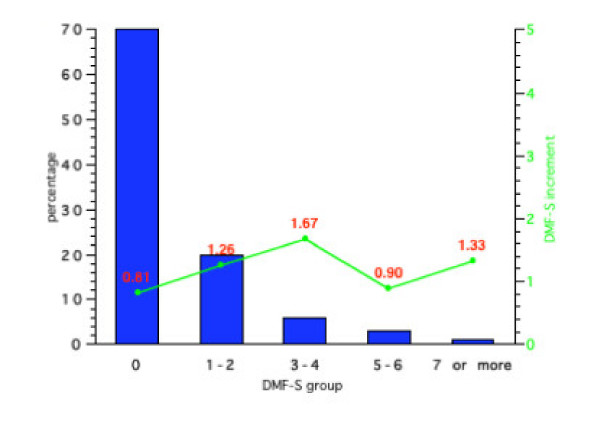
Initial percentage distribution and subsequent mean 4-year DMF-S increments for Group 4 (Initial DMF-S = 0.95).

Figures 1, 2, 3, 4 show the initial percentage distribution of caries and subsequent mean 4-year caries increment for 7 year-olds in the NPDDP programme according to fluoridation and fissure sealant status. At baseline of the children in Group 1 with an initial mean DMF-S score of 0.61, 70% had no caries while the comparable percentage with no caries for Group 2 who had an initial mean DMF-S of 0.85, was 67%. Twenty percent of the children in Group 1 had a DMF-S of 1 or 2, 6% a DMF-S of 3 or 4, and 3% a DMF-S of 5 or 6. Only 1% of children in this group had a DMF-S of 7 or 8. For Group 2 the respective figures were: 18% with a DMF-S of 1 or 2, 8% a DMF-S of 3 or 4, 4% a DMF-S of 5 or 6 and 2% a DMF-S of 7 or more.

For Group 3 (baseline DMF-S = 0.86), 67% had an initial DMF-S of 0, 19% 1 or 2, 8% 3 or 4, 4% 5 or 6 and 7% an initial DMF-S of 7 or more. For Group 4 (baseline mean DMF-S = 0.95) 65% had a DMF-S of 0, 20% a DMF-S of 1 or 2, 7% 3 or 4, 5% 5 or 6 and 3% a DMF-S greater than 7. No children in any of the groups had a DMF-S score of 9 or more.

Figures 1, 2, 3, 4 also show the mean caries increment for each of the sub-groups according to initial caries levels. For example, for those children with a DMF-S of 0 at the start of the 4-year programme in Group 1, the 4-year increment was 0.81, while for Group 4, the increment was 1.08. The highest increments were in Groups 2 and 3. For those children in Group 3 with an initial DMF-S of 5 or 6, the mean 4-year increment was 4.54

**Figure 5:**
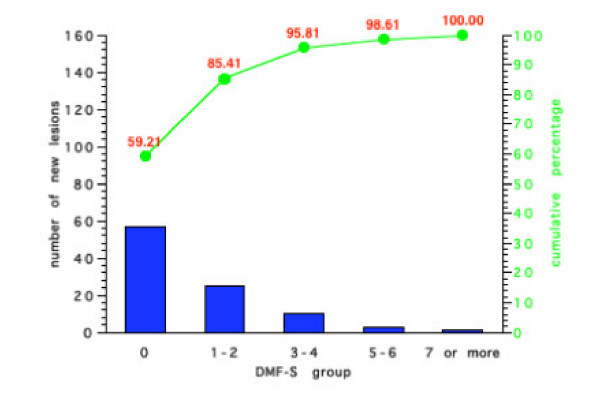
Number of new carious lesions per 100 children and cumulative percentage of lesions by grouped DMF-S score in Group 1 over a 4-year period.

**Figure 6:**
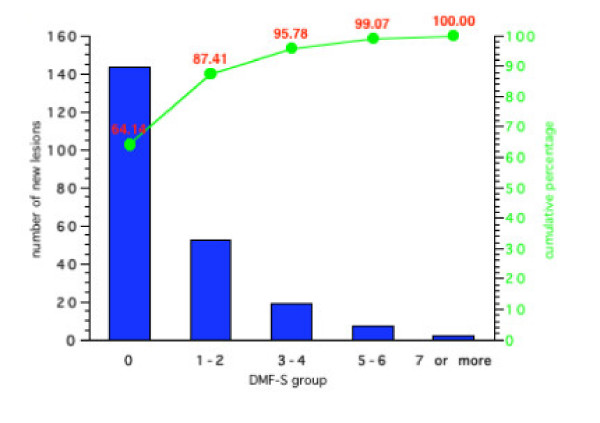
Number of new carious lesions per 100 children and cumulative percentage of lesions by grouped DMF-S score in Group 2 over a 4-year period.

**Figure 7:**
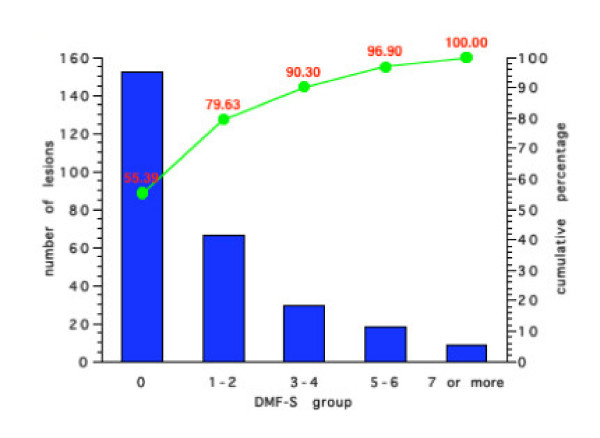
Number of new carious lesions per 100 children by grouped DMF-S score and cumulative percentage in Group 3 over a 4-year period.

**Figure 8:**
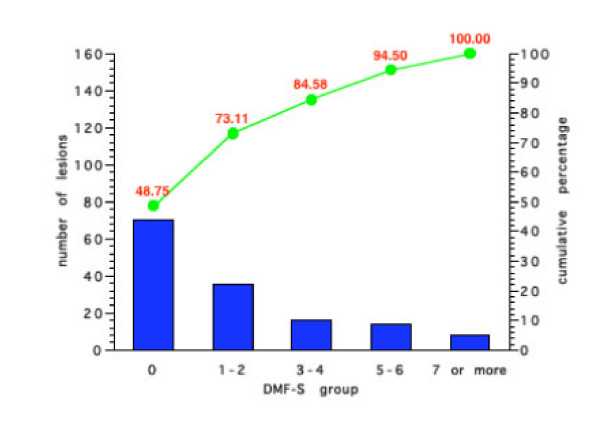
Number of new carious lesions per 100 children by grouped DMF-S score and cumulative percentage in Group 4 over a 4-year period.

Figures 5, 6, 7, 8 show the results for the four groups in terms of number of new carious lesions and cumulative percentage of the total number of lesions by initial DMF-S sub-group. The mean 4-year DMF-S increment of each group was multiplied by the percentage of individuals within the group. The results are expressed as the number of lesions per 100 children. For example, the mean DMF-S increment for the group of children in Group 1 with a baseline initial DMF-S of 0, was 0.81. This sub-group with a initial DMF-S of 0 constituted 70% of the whole Group 1. Thus the total number of lesions over a four-year period within this sub-group was 0.81 × 70, i.e. 57 lesions. For each Group's sub-groups, the total number of lesions was calculated by summing the number of lesions from each of the various initial DMF-S sub-groups. For example in Group 1 (Figure 5), the number of lesions over the 4 year period in the sub group with an initial DMF-S of 0 was 56.9, in the group with an initial DMF-S of 1–2, 25.1 lesions, in those with an initial DMF-S score of between 3–4, 10 lesions, 5–6, 2.7 lesions, and for those individuals with a DMF-S score of 7 or more, 1.3 lesions. The total number of lesions in the whole group was 97. Thus, the percentage of lesions accounted for by the sub group at an initial DMF-S of 0 was 56.91 divided by 97, i.e. 59.2%. Less than 2% of the new lesions that occurred in the sub-group at highest risk in Group 1, were in those with an initial baseline DMF-S score of 7 or more. Similar findings occurred in all the other three Groups. The sub-group of children at lowest risk, those with a baseline DMF-S of 0, accounted for the greatest percentage of new lesions and those at the highest risk accounted for the lowest percentage within the groups. For example, in Group 4, 48.7% of new lesions occurred in the sub-group of children with a baseline DMF-S score of 0, and 5.5% by the sub-group with a baseline DMF-S score of 7 or more.

## Discussion

These findings challenge the fundamental arguments used to justify the adoption of a high-risk strategy from a public health perspective. Namely, the main strategy should be directed at the smallish group with high baseline caries levels because they are at highest risk of future caries. As stated by Rose [9] when applied to general health and by Batchelor and Sheiham [2] for caries, the largest "... burden of ill health comes more from the many who are exposed to low inconspicuous risk than from the few who face an obvious problem". Burt [14], reviewing the concepts of risk as applied to dental public health, reinforced this approach arguing that the geographic targeting of caries preventive programmes should supplement population based approaches. Geographic targeting is one form of a directed population approach: an approach that uses socio-demographic or epidemiologic data to identify groups as opposed to screening for individuals who may benefit from the intervention [15].

Irrespective of the preventive regimes adopted, for all sub-groups, the majority of the new lesions were in those children who would have been classified as at lowest risk. Indeed, with the exception of those individuals in non-fluoridated areas and receiving sealants, over 50% of the total number of new lesions occurred in individuals with an initial DMF-S score of 0. For all preventive regimes, the contribution made by individuals with the highest initial grouped DMF-S score, those with 7 or more lesions, was less than 6% of the total new caries over a 4 year period. For example, for those individuals receiving fluoridated water and sealants, the contribution to the total number of lesions made by those with an initial DMF-S of 7 or more was less than 2%.

## Conclusion

The present study has shown that from a public health perspective, policy for caries preventive strategies should be based on a 'population' or 'directed population' approach.

## Pre-publication history

The pre-publication history for this paper can be accessed here:


